# Dose and strain dependent lethality of Usutu virus in an Ifnar^−/−^ mouse model

**DOI:** 10.1038/s44298-025-00089-x

**Published:** 2025-01-28

**Authors:** Johanna M. Duyvestyn, Eleanor M. Marshall, Peter J. Bredenbeek, Barry Rockx, Martijn J. van Hemert, Marjolein Kikkert

**Affiliations:** 1 Molecular Virology Laboratory, Leiden University Center for Infectious Diseases (LUCID), Leiden University Medical Center, Leiden, The Netherlands; 2 Department of Viroscience, Erasmus University Medical Center, Rotterdam, The Netherlands

**Keywords:** Virology, Viral pathogenesis

## Abstract

Usutu virus (USUV) is a mosquito-borne zoonotic flavivirus with a geographic range that has expanded over recent years. Maintained in a transmission cycle between mosquito vectors and avian reservoirs the virus can cause large seasonal outbreaks in bird populations, but spillover into mammalian hosts has also been reported. While usually mild or asymptomatic in humans, neurological disorders are increasingly observed, which has boosted interest and the need for better understanding of the pathogenesis of various USUV lineages. In this study we inoculated interferon α/β receptor knockout (Ifnar^−/−^) mice with decreasing doses of USUV, monitoring symptoms and survival to determine a less lethal dose, and we directly compared isolates from three different viral lineages. We found that a Dutch isolate of USUV Africa-3 lineage is lethal at a dose of 20 pfu per mouse, which is considerably lower than what was anticipated based upon the literature. A Europe-2 strain showed an even higher virulence in this mouse model, compared to strains from Africa-3 and Europe-3 lineages—though this was not reflected in in vitro studies. These results enhance our understanding of the pathogenicity of different USUV strains and provide guidance for the use of low doses for inoculation in an Ifnar^−/−^ animal model.

## Introduction

Usutu virus (USUV) is an emerging zoonotic arbovirus within the Japanese encephalitis virus complex in the genus *Orthoflavivirus*. Initially discovered in South Africa in 1959, the geographical range of USUV outbreaks has extended across the Middle East and Europe, and there are indications of the virus now becoming endemic in some European regions^1^. Phylogenetic analyses have indicated multiple introductions of distinct USUV strains into Europe, resulting in the circulation of a diverse set of strains that are categorised into eight different lineages, named Africa (Af) 1–3 and Europe (Eu) 1–5^2^. The increasing geographic range of vector mosquitoes caused by rising global temperatures further exacerbates the risks of infection with USUV and other clinically relevant and emerging arboviruses^3,4^.

USUV is primarily circulating between mosquitoes of the Culex genus and avian reservoir hosts. Mammals including humans, horses, and rodents have been identified as incidental hosts. In certain bird species, such as black birds, infection can lead to high mortality rates causing large population declines^5,6^. In humans, USUV infections are usually asymptomatic, or cause mild symptoms. However, symptoms such as fever, rash, and headache can occur, and in rare cases the development of severe neuroinvasive disease, predominantly in immunocompromised individuals, is observed^6^.

Given its rising prevalence, and the increasing trend in epidemics caused by arboviruses in general, investigating USUV in relevant models is essential to further our understanding of virulence and other risk factors in order to develop preventive measures against this virus^3^. Although there is rapidly expanding development of animal-free models to study these viruses (providing both an ethically preferrable option as well as potential for more accurate translation into humans), they are not yet feasible for investigating all aspects of the infection or the efficacy of vaccines and antivirals. Pre-clinical animal models are therefore still the gold standard for orthoflavivirus vaccine and drug research^7,8^. While some avian models have been developed and have provided insights into USUV pathology in reservoir species, birds present many more logistical challenges for standard laboratory research than well-developed inbred mouse models^9^.

Immunocompetent mammals have variable but generally low susceptibility to USUV, unless neonatal or suckling animals are used, which are more susceptible. Immunocompromised models however display pronounced signs of USUV disease, with high mortality rates, although the degree of lethality and the rate of disease progression vary widely. Several factors, including the specific animal model, the strain of the virus, the viral dose used, and the inoculation method all influence the measured outcomes^9^. Table 1 gives an overview of this variation in studies that have employed immunocompromised mouse models to study USUV.

**Table 1:** Summary of earlier studies assessing USUV lethality in immunocompromised mouse models

Study	Model	Age/Sex	Inoculation/Dose	Lineage + isolate (Host species)	Survival Results
Segura Guerrero et al.^10^	IFN α/β/γ receptor^−/−^ (129)	8–14 wk Male	IP 1 × 10^6^ pfu	Eu-* strain V18/BH65/11-02-03(TM)	100% Lethal Day 2–3
			IP 1 × 10^5^ pfu		100% Lethal Day 3
			IP 1 × 10^4^ pfu		70% Lethal Day 4
			IP 1 × 10^3^ pfu		100% Lethal Day 4–5
			IP 1 × 10^2^ pfu		75% Lethal Day 4
			IP 1 × 10^1^ pfu		70% Lethal by Day 9
Bates et al.^11^	CD-1 (anti-Ifnar1)	3 wk Male	Inj.* 1 × 10^5^ pfu	Af-3 UG09615/UG-2012 (Cx)	40% Lethal Day 10
				Af-3 TMNetherlands-2016/NL-2016 (TM)	Non-lethal.
Salgado et al.^12^	CD-1 (anti-Ifnar1)	8 wk Male + Female	SC Footpad 1 × 10^3^ pfu	Af-2 HU10279-09/SP-2009 (Cx)	No morbidity or weight loss in either strain
				Af-3 UG09615/UG-2012 (Cx)	
Martín-Acebes et al.^13^	Ifnar^−/−^ (129)	6 wk Male + Female	IP 1 × 10^4^ pfu	Af-2 Biotec (Vero-derived SAAR1776)	90% Lethal Day 5–10
Clé et al.^14^	Ifnar^−/−^ (B6)	8–12 wk Male	IP 1 × 10^4^ TCID50	Af-2 Rhône2705/FR-2015 (TM)	100% Lethal Day 6
Constant et al.^15^	Ifnar^−/−^ (*)	Adult	Subdermal 1 × 10^3^ TCID50	Eu-2 TE20421/IT-2017 (TM)	100% Lethal Day 3–5 (Show neuroinvasion).
Kuchinsky et al.^16^	Ifnar^−/−^ (B6)	8 wk Male	SC footpad 1 × 10^3^ pfu	Af-2 SAAR-1776/SA-1959 (Cx)	100% Lethal Day 5–6
				Af-2 HU10279-09/SP-2009 (Cx)	100% Lethal Day 5–6
				Af-3 DakPM173701/SE-2003 (Cx)	100% Lethal Day 4–5
				Af-3 UG09615/UG-2012 (Cx)	100% Lethal Day 5–6
				Af-3 TMNetherlands 2016/NL-2016 (TM)	10% Lethal Day 7
**Study**	**Model**	**Age/Sex**	**Inoculation/Dose**	**Lineage(s)**	**Results**
Salgado et al.^12^	Ifnar^−/−^ (B6)	10–18 wk Male + Female	SC Footpad 1 × 10^3^ pfu	Af-2 HU10279-09/SP-2009 (Cx)	100% Lethal Day 7
				Af-3 UG09615/UG-2012 (Cx)	100% Lethal Day 5 (SP-2009 shows lower viremia titres)
Bates et al.^11^	Ifnar^−/−^ (B6)	3 wk Male	Inj.* Footpad 2 × 10^5^ pfu	Af-3 UG09615/UG-2012 (Cx)	100% Lethal Day 3
				Af-3 TMNetherlands-2016/NL-2016 (TM)	100% Lethal Day 6
This study	Ifnar^−/−^ (B6)	Adult Male + Female	SC Hind limb 1 × 10^2^ pfu	Eu-2 Bologna-2009/IT-2009 (Hu)	100% Lethal Day 4
				Eu-3 AS201700077/NL-2017 (TM)	100% Lethal Day 5
				Af-3 TMNetherlands/NL-2016 (TM)	100% Lethal Day 6
			2 × 10^1^ pfu	Af-3 TMNetherlands/NL-2016 (TM)	90% Lethal Day 8

Models: B6 -C57BL/6J mice, 129 - 129S1/SvImJ mice. Injection Methods: IP – Intraperitoneal, SC – Subcutaneous, Inj. – injection, anti-Ifnar1 – Transient treatment with anti-Ifnar1 antibody. Host species: TM (*Turdus merula*/black birds), Cx (*Culex* species mosquitoes). Other: *Not specified.

Establishing a dose of USUV that is only just lethal could make comparative virulence studies less difficult to interpret, by slowing disease progression and allowing small differences in virulence to be distinguished. From the current literature, it is not easy to determine such a threshold dose in an Ifnar^−/−^ model. A single study that compared infection with different doses of an Eu-3 lineage USUV in AG129 (IFN α/β/γ receptor−/−) mice showed that decreasing doses of the virus increased survival time and decreased lethality, although without a clear linear correlation^10^. No such dose comparison studies have been performed in Ifnar^−/−^ mice. Earlier studies in Ifnar^−/−^ mice have used doses of 1 × 10^3^–1 × 10^4^ pfu or TCID50 per mouse, in most cases resulting in 100% lethality between days 4–6 post-infection^11–15^. An exception to this was the Af-3 lineage TM-Netherlands-2016 strain which showed a dramatically reduced mortality in both Ifnar^−/−^ and transiently immunocompromised CD-1 mouse models^11,16^.

Additionally, the difference in virulence between USUV lineages is not well understood. Table 2 gives an overview of studies comparing USUV strains from the different lineages. A few trends can be seen in the data - strains from the Eu-2 lineage appear to be more virulent^17–19^, and studies comparing Af-3 and Af-2 strains suggest that virulence within one lineage can vary as much as virulence between the lineages^11,16^. However, ranking lineages by their virulence can be inconsistent and challenging, even when using similar animal models^9^, and it is not clear whether the differences can be generalised to a particular lineage or are strain-specific (Table 2). For example, Cle et al. found that percent survival did not always correlate with average survival time, and which strain was more pathogenic differed depending on the model used, though Eu-2 strains were consistently more lethal^18^. Moreover, there are additional complications in assessing the literature, as lineage nomenclature can be confused with references to the location of isolation. To obtain a better insight into the virulence of USUV lineages, more studies are needed that systematically and directly compare the pathogenicity of different USUV strains in the same animal model.

**Table 2:** Summary of studies comparing USUV strains

Host	Model		Isolate comparison summary	Reference
Mosquito	In vitro	C6/36	Af-3^UG-2012^ > Af-3^NL-2016^	Bates et al.^11^
		C6/36 + coinfection	Eu-2^IT-2009^ > Af-3^NL-2016^	van Bree et al.^19^
	In Vivo	*Culex quinquefasciatus*	Af-3^NL-2016^ > Af-3^UG-2012^	Kuchinsky et al.^31^
		*Culex pipiens* co-infection	Eu-2^IT-2009^ > Af-3^NL-2016^	van Bree et al.^19^
Avian	In vitro	DF-1	Af-3^NL-2016^ = Af-2^SP-2009^ > Af-3^UG-2012^ = Af-2^SA-1959^	Kuchinsky et al.^32^
		DF-1	Eu-2^IT-2009^ = Af-3^NL-2016^	van Bree et al.^19^
		DF-1 co-infection	Eu-2^IT-2009^ > Af-3^NL-2016^	van Bree et al.^19^
		Chicken Chorioallantoic Membrane (CAM)	Eu-3^BE-Seraing/2017^ > Eu-1^Vienna2001^ > Eu-2^UR-10-Tm^ = Af-3^BE-Grivegnee/2017^	Benzarti et al.^34^
	In Vivo	Chicken embyros	Eu-1^Vienna2001^ = Eu-2^UR-10-Tm^ = Eu-3^BE-Seraing/2017^ = Af-3^BE-Grivegnee/2017^	Benzarti et al.^34^
		House sparrows	Af-3^NL-2016^ > Af-3^UG-2012^	Kuchinsky et al.^31^
		ISA chickens	Af-3^NL-2016^ > Af-3^UG-2012^	Kuchinsky et al.^32^
		LAS chickens	Af-3^NL-2016^ = Af-3^UG-2012^	Kuchinsky et al.^32^
		Avian Pathology samples	Eu-3* **=** Af-3^a^	Giglia et al.^20^
Mammalian	In vitro	Vero CCL-81	Af-3^NL-2016^ = Af-3^UG-2012^	Bates et al.^11^
		Vero E6	Eu-2^IT-2009^ = Af-3^NL-2016^	van Bree et al.^19^
		Vero E6 co-infection	Eu-2^IT-2009^ > Af-3^NL-2016^	van Bree et al.^19^
		Epidermal Keratinocytes	Eu-2^IT-2017^ > Af-2^FR-2015^ > Eu-5^DE-2016^ > Af-3^FR-2018^	Vouillon et al.^18^
		Primary Human CNS cell lines	Eu-2^IT-2017^ > Eu-3^FR-2015^ > Af-3^FR-2018^ > Eu-5^DE-2016^ > Eu-1^AT-2001^ > Af-2^FR-2015^	Cle et al.^17^
		Blood Brain Barrier model	Eu-2^IT-2017^ > Af-3^FR-2018^	Cle et al.^17^
	In Vivo	Swiss mice (Weanling)	Af-2^SAAR-1959^ > Af-3^CAR-1981^ = Af-2^ROD259266^	Diagne et al.^33^
		Swiss mice (Neonatal)	Eu-2^IT-2017^ > Eu-5^DE-2016^ > Af-3^FR-2018^ > Eu-1^AT-2001^ > Eu-3^FR-2015^ > Af-2^FR-2015^	Cle et al.^17^
		129/Sv mice	Eu-3^BE-Seraing/2017^ = Af-3^BE-Grivegnee/2017^	Benzarti et al.^35^
		IFNAR^−/−^ mice	Af-3^SE-2003^ > Af-3^UG-2012^ = Af-2^SA-1959^ = Af-2^SP-2009^ > Af-3^NL-2016^	Kuchinsky et al.^16^

^a^Not specified.

In this study we have compared two Dutch USUV isolates from lineages that have been implicated in outbreaks among birds in the Netherlands (lineage Af-3 and lineage Eu-3)^20^, and an isolate from the clinically relevant Eu-2 lineage. We assessed replication kinetics of these strains in relevant cell lines and compared their pathogenicity in an Ifnar^−/−^ mouse model. To establish a model more likely to identify subtle differences in virulence we first optimised a subcutaneous dose of USUV, at which we observed ~90% lethality, using the Af-3 strain (Netherlands 2016, Af-3-NL). Our characterisation of USUV infections in the Ifnar^−/−^ mouse model as described here will form the basis for further studies into USUV pathogenesis.

## Methods

### Viruses

The USUV virus stocks, as listed in Table 3, were received from Erasmus Medical Center Rotterdam, The Netherlands, and were passaged twice on Vero CCL-81 cells at 37 °C, 5% CO_2_ in Dulbecco’s modified Eagle’s medium (DMEM, Gibco) supplemented with 8% foetal calf serum (FCS, Capricorn Scientific), and 100 units/mL of streptomycin/penicillin (Sigma-Aldrich), 1% sodium bicarbonate (Gibco) and 2mM L-glutamine (Sigma-Aldrich). Infectious virus titre was determined by plaque assay on BHK-21J cells.

**Table 3:** Virus isolate details

Virus isolate^a^	Usutu lineage	Strain Details (Host, Location, Year)	Passage	GenBank accession no.	Reference
Af-3-NL	Africa 3	AS201600045 T. merula Netherlands 2016	2	MH891847	^36^
Eu-2-IT	Europe 2	Homo sapiens Bologna 2009	3	HM569263.1	^37^
Eu-3-NL	Europe 3	AS201700077 T. merula Netherlands 2017	2	MN122189	^38^

^a^Denotes virus names as used in this text.

### Recombinant USUV cDNA clone

Mutant recombinant USUV cDNA clones were built by a TAR recombineering protocol in yeast, adapted to our research lab from the method described in^21^. Briefly, overlapping fragments of the USUV genome were amplified by PCR using a recombinant Af-3-NL clone as a template (Nelemans et al. manuscript in preparation, GenBank Accession no. PQ041659). The fragment containing the envelope (E) region was cloned into the pCR™8/GW/TOPO vector (Thermofisher) and site-directed mutagenesis was used to insert the required nucleotide change (Primers in Supplementary Table 1a). The PCR products were purified and assembled into the pCC1BAC-his3 vector by transformation-associated recombination (TAR) *in S. cerevisiae*. Following colony screening using a multiplex PCR targeting all the assembly junctions, the DNA was purified and transformed into *E. coli* for large-scale plasmid extraction. Sanger sequencing of the plasmid was performed to confirm the presence of the mutation. To launch the virus, linearised plasmid was reverse transcribed, and the purified RNA was electroporated as described previously into BHK21-J cells^22^. The supernatant was harvested after 4 days and used to inoculate Vero CCL-81 cells in order to grow a p1 stock. The full genome was analysed by NGS to confirm the presence of the mutation and absence of other (undesired) mutations.

### Cell lines

All cells were maintained at 37 °C in a 5% CO_2_ incubator. Vero CCL-81 cells (VeroMM-2, LUMC cell line collection) and A549 cells (LUMC cell line collection) were cultured in DMEM supplemented with 8% FCS and 100 units/mL of streptomycin/penicillin. BHK21-J cells^23^ were cultured in Glasgow’s MEM (GMEM, Gibco) supplemented with 8% FCS, 10% tryptose phosphate broth (Gibco), 10 mM HEPES (Lonza), and 100 units/mL of streptomycin/penicillin (Sigma-Aldrich). Human neuroblastoma (neuron-like, SK-N-SH, Sigma-Aldrich) were maintained in Eagle minimal essential medium (EMEM, Lonza) supplemented with 10% foetal bovine serum (FBS, Sigma-Aldrich), 100 IU/ml penicillin (Lonza), 100 μg/ml streptomycin (Lonza), 2 mM glutamine (Lonza), 1% sodium bicarbonate (Lonza), sodium pyruvate (Sigma) and 1 × nonessential amino acids (Capricorn scientific), and were used until passage 20. Human astrocytes (HA, Sciencell) were maintained in the recommended Astrocyte medium (AM, Sciencell) prepared as per the manufacturer’s instructions, and were used until passage 10. Human brain microvascular endothelial cells (BMECs, Cell systems) were maintained in MV2 medium (Promocell) prepared as per the manufacturer’s instructions and were used until passage 12.

### Viral growth kinetics

Cells were grown to 80% confluency in multi-well plates. Medium was removed and cells were infected at an MOI 0.1 for 1 h at 37 °C. After removal of the inoculum, cells were washed gently three times with PBS before adding viral growth media (see above). Plates were incubated at 37 °C and supernatant/medium was collected at the specified timepoints, and for Vero CCL81 and A549 experiments, cells were lysed in GITC buffer (3 M guanidine-thiocyanate, 2% N-lauroylsarcosine, 50 mM Tris-HCl pH 7.6, and 20 mM EDTA).

### Virus quantification

For determining viral RNA copy numbers, RNA from supernatants or cell lysates was isolated using the Bio-on-Magnetic-Beads method^24^. Reverse transcriptase quantitative PCR (RT-qPCR) was performed with TaqMan Fast Virus 1-step master mix (Thermofisher) in a CFX384 Touch real-time PCR detection system (Bio-Rad) using a programme consisting of 3 min at 95 °C and 30 s at 60 °C followed by 40 cycles of 10 s at 95 °C, 10 s at 60 °C and 30 s at 72 °C. Samples were run alongside a reference standard to determine copies/ml, and samples from selected time points were additionally titrated by TCID50 assay to confirm the correlation to infectious particles. For analysis, the Ct cut-off was set to 35 cycles. The primers used for RT-qPCR are listed in Supplementary Table 1b.

Plaque assays were performed on BHK21-J cells at 70% confluency in 12-well or 6-well clusters. Samples were tenfold serially diluted in DMEM–2% FCS and used to inoculate the monolayer for 1 h at 37 °C. Inoculum was replaced with an overlay of DMEM, 1.2% Avicel (FMC BioPolymer), 2% FCS, 50 mM HEPES, and antibiotics. Cells were fixed with 3.7% formaldehyde in PBS at day 4 post-infection and plaques were visualised using crystal violet staining. The detection limit of the assay was 40 pfu/ml (analysis of the 1:10 sample dilution).

For TCID50 titration tenfold serial dilutions of culture supernatants were inoculated onto a semiconfluent monolayer of Vero cells in a 96-well plate (2.3 × 10^4^ cells/well). Cytopathic effect (CPE) was scored at 6 days post-infection. Virus titres were calculated using the Spearman-Kärber method^25^. An initial 1:10 dilution of supernatant resulted in a detection limit of 31.6 TCID50/ml.

### Cytotoxicity assay

Lytic cell death was indirectly measured by lactate dehydrogenase (LDH) release in cell culture supernatant using the CytoTox 96 nonradioactive cytotoxicity assay kit (G1780, Promega). The assay was conducted according to the manufacturer’s instructions and absorbance was measured at 490 nm with an EnVision multiplate reader (PerkinElmer). Values were normalised using cells treated with 1% triton-X100 (100% LDH release).

### RT-qPCR to monitor host cell responses

RNA was isolated as described above from cell lysates harvested at 24 h, and reverse-transcribed into cDNA using the RevertAid H Minus reverse transcriptase (Thermo Scientific) and random hexamers. Real-time quantitative PCR was performed with iQ SYBR green Supermix (Biorad) in a CFX384 Touch real-time PCR detection system (Bio-Rad) using the following programme: 3 min at 95 °C and 30 s at 60 °C followed by 40 cycles of 10 s at 95 °C, 10 s at 60 °C and 30 s at 72 °C. Gene expression was quantified by standard curve, normalised to expression of RPL13a as a house-keeping gene and the fold change compared to uninfected control samples was calculated. Statistical analysis was calculated by the unpaired two-tailed Student’s *t*-test. The primers used for RT-qPCR are listed in Supplementary Table 1c.

### Mouse studies

Ifnar^−/−^ mice in a C57BL/6 background (B6(Cg)-Ifnar1<tm1.2Ees>/J) were bred and maintained in pathogen-free facilities at the LUMC Central Animal Facility (PDC) at 20–22 °C, a humidity of 45–65% RV and a light cycle of 6:30 h–7:00 h sunrise, 07:00 h–18:00 h daytime and 18:00 h–18:30 h sunset. Mice had access to water and food ad libitum and were provided with cage enrichment. Age-matched male and female mice were arranged in groups of 8 (virus inoculation) or 3 (controls) and acclimated to the experimental facility for 7 days. Mice (6–8 weeks expt1, 12–15 weeks expt2, 9–10 weeks expt3) were inoculated with 100 µl of virus in DMEM (Gibco), or DMEM alone via sub-cutaneous (SC) injection into the hind limb. SC injection was selected as it is more representative of a mosquito infection than IP administration, but we found it to be more consistent to perform than ID inoculation. Hind limb site was selected to minimise the time that animals were under anaesthetic and reduce discomfort after the inoculation. Doses ranging between 2 × 10^1^ and 5 × 10^4^ pfu/ml were used for USUV-Af-3 dose determination experiments, and 1 × 10^1^ pfu/ml was used for lineage comparison studies. Plaque assays of the virus inocula were performed on BHK21-J cells to confirm that the mice were inoculated with the intended dose for each animal experiment (Supplementary Table 2).

Mice were weighed and monitored daily for the following clinical symptoms; activity, coat condition, hind limb function, ocular discharge. Sera from tail vein bleeds were collected on alternating days. Upon reaching humane endpoints (in agreement with vet), or at the end of the experiment, mice were euthanized by CO_2_ and a final serum sample was taken by heart puncture. Surviving animals and mock-infected mice were harvested at day 14 or least 5 days after the last infected animal succumbed. Tissue samples from heart, liver, spleen, and kidney, as well as brain sections were dissected, weighed and placed in viral transport medium (VTM, MEM without L-glut and HEPES Buffered, 100 units/mL of streptomycin/penicillin, Amphotericin B, Gentamycin 10% Glycerol) then frozen for further processing (Note: brain half was further dissected into either olfactory bulb, frontal lobe, cerebellum, cortex and brain stem, or as forebrain and hindbrain).

### RNA isolation and viral load determination from mouse tissue

Serum samples from mouse bleeds were inactivated with 0.2% triton-x, diluted 1:20 and used directly for RT-qPCR. Tissue samples in VTM were defrosted, homogenised by pulsation with mixed sizes of acid-washed glass beads 425–600 μm (Sigma-Aldrich) and 3 mm (VWR international) in a PRECELLYS® 24 Tissue homogeniser (Bertin Instruments), then centrifuged to obtain a clean supernatant. RNA was isolated and viral load was determined by RT-qPCR as described above. Samples were run alongside a USUV reference standard to determine pfu equivalents or USUV genome copy numbers per gram of tissue. A subset of animal samples from each group was analysed by plaque assay to confirm the correlation between RT-qPCR measured copy numbers and infectious particles.

### Ethics declaration

All experiments involving animals were approved by the Animal Experiments Committee of the LUMC and performed according to the recommendations and guidelines set by the LUMC, the Dutch Experiments on Animals Act, and were in strict accordance with EU regulations (2010/63/EU).

### Statistical analysis

Statistical analyses were performed in GraphPad Prism (version 9). All data are represented as mean ± SEM unless stated otherwise. Survival experiments were analysed using log-rank (Mantel-Cox) test. Viral titres in mouse sera were analysed using a one-way ANOVA for each time point. Viral titres of mouse tissues were analysed using unpaired t-test corrected for multiple analysis by two-stage step-up approach (Benjamini, Krieger and Yekutieli).

## Results

### Low doses of USUV Af-3-NL cause lethal infections in Ifnar^−/−^ mice

**Fig. 1:**
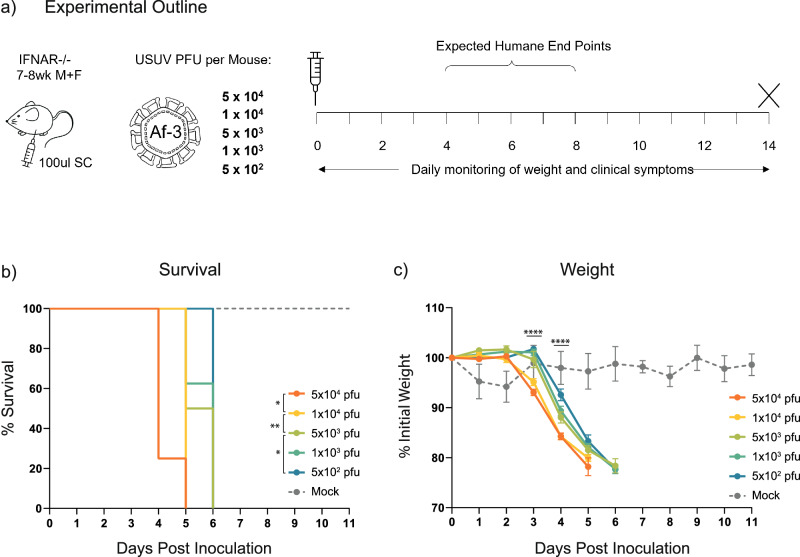
USUV Af-3-NL infected mice show rapid disease progression and lethality at doses as low as 500 pfu. **a** Ifnar^−/−^ mice were inoculated subcutaneously with USUV Af-3-NL, with doses ranging from 5 × 10^4^ to 5 × 10^2^ pfu/mouse (*n* = 8) or with DMEM (*n* = 3). Animals were euthanized when they reached a humane endpoint. **b** Survival rates for each of the experimental groups. Statistical analysis was performed using the log-rank (Mantel-Cox) test. **c** Daily weight loss measured as a percentage of initial weight for each of the experimental groups showing mean ± SEM. Statistical analysis was performed using a one-way ANOVA for each time point. *P < 0.05, **P < 0.01, ****P < 0.0001.

To develop a model capable of distinguishing subtle differences in USUV virulence, we hypothesised that using a lower infection dose would decelerate disease progression and increase the average survival time as well as the timespan over which symptoms can be monitored. Less virulent strains would then be easier to discriminate based on lethality. In an attempt to establish a minimal lethal dose of Af-3-NL in an Ifnar^−/−^ mouse model, we selected a dose range, based on the literature, expected to yield outcomes that range from 100% lethality to 100% survival. Af-3-NL virus inoculum was subcutaneously administered into Ifnar^−/−^ mice with doses ranging from 5 × 10^4^ to 5 × 10^2^ pfu/mouse, while control animals received DMEM medium (Fig. 1a).

We found that while lethality was delayed in a dose-dependent manner, the lowest dose of the virus still resulted in 100% lethality by day 6 post-inoculation (Fig. 1b). In contrast, all mock-injected mice survived with the only notable symptom being a mild weight loss on the first day (Fig. 1c) from which they recovered quickly. While onset was delayed for the lower dosed animals, disease progression occurred at a similar rate. A measurable decrease in weight correlated with the onset of other observed clinical symptoms, and humane end point (HEP) was reached ~1–2 days after this time (Fig. 1c). We initially observed a limp in the injected hind limb, a reduction in overall activity, and a white ocular discharge in one or both eyes. These symptoms progressed to a point where animals were unable to open their eye/s, were hunched, and had ruffled fur (the latter was more noticeable in male than female mice) at which point the animals were sacrificed (Table 4). At the 5 × 10^2^ pfu dose we also observed a single ataxic animal, nearing HEP, exhibiting side-to-side swaying while being inactive and in a hunched position.

**Table 4:** Median day of onset of clinical symptoms in USUV-dosed mice

Clinical Symptom	Median Day of Symptom Onset per Group				
	5 × 10^4^ pfu	1 × 10^4^ pfu	5 × 10^3^ pfu	1 × 10^3^ pfu	5 × 10^2^ pfu
Reduced activity^a^	4	4	4	5	5
Hunched posture	5	5	5	6	6
Limping^b^	4	4	4	5	5
Ocular discharge^c^	4	5	5	5	6

^a^Score when animals are no longer running around cage unprompted.

^b^Limp developed in the inoculated hind limb.

^c^White discharge in one or both eyes resulting in partial or full closure of eye.

**Fig. 2:**
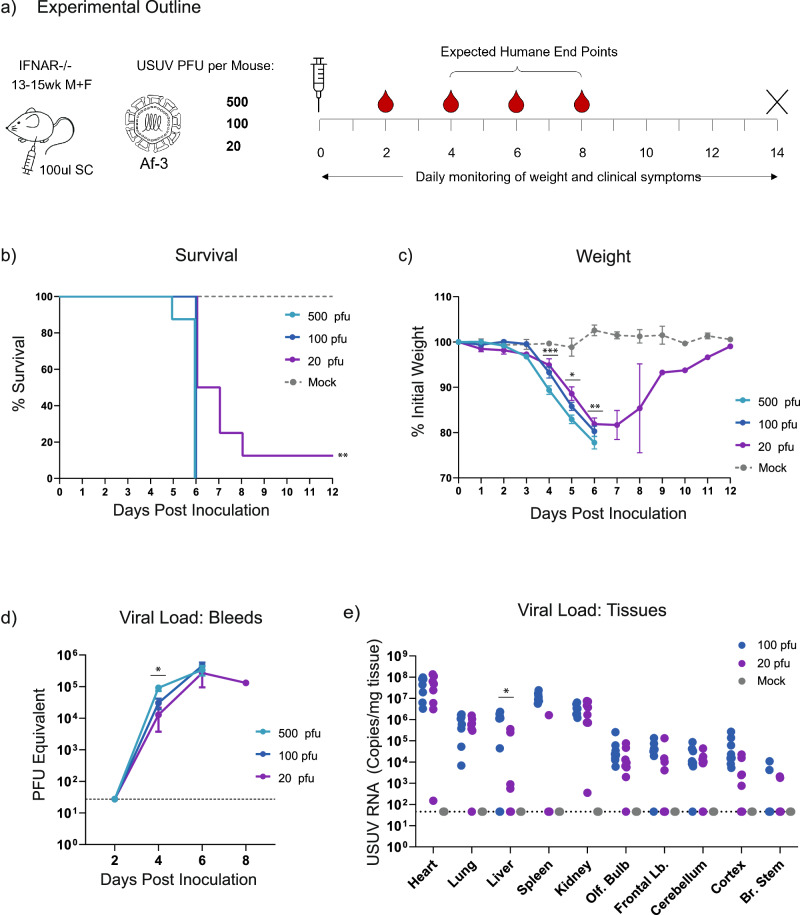
USUV Af-3-NL retains 90% lethality at doses as low as 20 pfu per mouse. **a** Ifnar^−/−^ mice were inoculated subcutaneously with USUV Af-3-NL at a dose of 500, 100, or 20 pfu/mouse (*n* = 8 per group) or with DMEM (*n* = 3). Mice were weighed daily, and serum was collected on alternating days. Animals were euthanized when they reached a humane endpoint, final bleeds were taken by heart puncture and relevant tissues were harvested. **b** Survival rates for each of the experimental groups. Statistical analysis was performed using the log-rank (Mantel-Cox) test. **c** Daily weight loss measured as a percentage of initial weight for each of the experimental groups showing mean ± SEM. Statistical analysis was performed using a one-way ANOVA for each time point. **d** Mean (±SEM) viral load of sera measured by RT-qPCR, using serial dilutions of USUV reference standard to determine pfu equivalents. Statistical analysis was performed using a one-way ANOVA for each time point. **e** USUV RNA copies/g of homogenised heart, lung, liver, spleen kidney and brain (dissected into olfactory bulb, frontal lobe, cerebellum, cortex and brain stem) tissues harvested at humane end point or end of experiment (day 12) measured by RT-qPCR using an USUV reference standard. Statistical analysis was performed using unpaired *t*-test corrected for multiple analysis. Limit of detection is represented as dotted grey line in (**d**, **e**). * *P* < 0.05, **P < 0.01, ***P < 0.001.

While the above results correlated with studies using other USUV strains, it contrasted with the lack of lethality observed by Kuchinsky et al. for this specific strain^16^ (Table 1). After confirming that the titre of the virus stock that we used for the inoculum was correct (by plaque assay and TCID50 assay), we repeated this experiment using even lower doses of virus, i.e. 100 and 20 pfu/mouse. We also included the 5 × 10^2^ (500) pfu dose used in the initial experiment to control for the possible impact of the increased age of the mice in these groups (Fig. 2a). Lethality in the groups that received 500 pfu was in line with our expectations based on the first experiment, with all mice reaching HEP by day 6. Disease progression and weight loss also followed the earlier observed trend. Mice inoculated with 100 pfu virus exhibited a similar disease progression and lethality, with slightly reduced weight loss. However, mice given the 20 pfu dose exhibited a lag in disease progression, and a single animal recovered from the infection (Table 5, Figs. 2b, c). We again observed a side-to-side sway in hunched mice nearing HEP, in a single 500 pfu-dosed mouse, and in three of the 20 pfu-dosed animals. These results indicated that 20 pfu is a sufficient lethal dose (approximately LD90, though we did not try lower doses to accurately define this) for USUV Af-3-NL in this Ifnar^−/−^ mouse model.

**Table 5:** Median day of onset of clinical symptoms in low dose USUV mice

Clinical Symptom	Median Day of Symptom Onset per Group		
	500 pfu	100 pfu	20 pfu
Reduced Activity^a^	5	6	6
Hunched Posture	5	6	7
Limping^b^	4.5	5	6
Ocular discharge^c^	5	6	6

^a^Score when animals are no longer running around cage unprompted.

^b^Limp developed in the inoculated hind limb.

^c^White discharge in one or both eyes resulting in partial or full closure of eye.

To further characterise the USUV infection at these reduced doses, we determined the viral loads in sera collected on alternating days (Fig. 2d), and in tissues harvested at the time of sacrifice (Fig. 2e). We detected no measurable virus in the blood at day two, but by day four, serum titre differed significantly in a dose-dependent manner (Fig. 2d). Animals infected with 500 pfu virus produced titres approximately three-fold higher than those that had received 100 pfu virus, and six-fold higher than those inoculated with 20 pfu virus. However, as animals neared the HEP, the peak viral titres across all dosage groups converged. Similarly, in the tissue samples harvested at HEP, the 20 pfu dose did not result in significantly lower viral loads than the 100 pfu dose, with the exception of the samples extracted from the surviving mouse (shown in Supplementary Fig. 1a–c).

### The E293K mutation in the envelope protein does not explain differences in pathogenicity between two otherwise genetically identical USUV Af-3 isolates

The highly pathogenic phenotype of our Af-3 isolate in mice contrasted with findings from an earlier study that found this strain to be attenuated compared to other strains^16^. When we compare the sequences, our isolate (Genbank accession number MH891847) contains a glutamic acid at position 293 in the E protein (Supplementary Fig. 2a) rather than a lysine in the published sequence from Kuchinsky et al. (Genbank accession number MN81349). Single amino acid changes can cause strongly attenuating phenotypes^26^, and we, therefore, hypothesised that this particular amino acid change could explain the diverging observations. We introduced the (charge reversing) E293K mutation into the coding sequence of the E protein in our recombinant USUV Africa-3 cDNA clone using site-directed mutagenesis and TAR recombineering, to assess whether this would affect pathogenicity of this isolate.

**Fig. 3:**
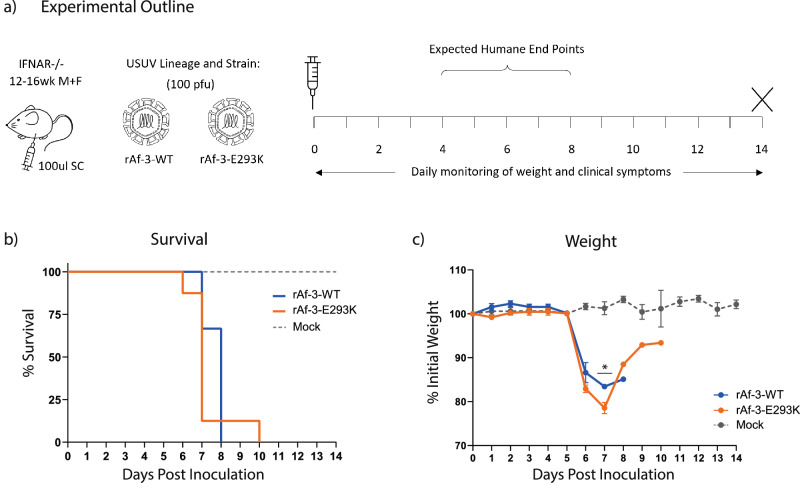
The E293K mutation in the E protein of USUV Af-3 does not lead to attenuation in mice. **a** Ifnar^−/−^ mice were inoculated subcutaneously with 100 pfu of rAf-3-WT (*n* = 8), rAf-3-E293K (*n* = 8) or with DMEM (*n* = 3), weighed daily and tail bled on alternating days. **b** Survival rates for each of the experimental groups. Statistical analysis was performed using the log-rank (Mantel-Cox) test. **c** Daily weight loss measured as a percentage of initial weight for each of the experimental groups showing mean ± SEM. Statistical analysis was performed using a one-way ANOVA for each time point.

Replication kinetics of the mutant virus were compared to the isogenic wild type in Vero CCL-81 cells, and we found no significant difference (Supplementary Fig. 2b). We then compared our wild type Af-3 clone and the mutant side-by-side in our Ifnar^−/−^ mouse model (Fig. 3a). Surprisingly, there was no significant difference in the survival of the mice infected with the Af-3-E293K mutant virus compared to those infected with rAf-3-WT virus (Fig. 3b). The mutant virus-infected mice succumbed slightly more rapidly, and showed greater weight loss (Figs. 3b, c and Supplementary Fig. 2c). A single Af-3-E293K infected mouse did show signs of recovery, demonstrating weight gain over several days, as well as increased activity, with healthy coat and minimal ocular discharge (Fig. 3c). However, several days later, the mouse lost the use of both hind limbs and needed to be euthanised.

The recombinant Af-3-E293K virus was analysed by NGS after harvesting from cell culture which confirmed the presence of the mutation (Supplementary Fig. 2d). We found that the clone contained a single additional mutation, NS2a-L18Q, but since there was no phenotypic difference with the wild type virus we did not look into this further.

### USUV strain-specific replication kinetics vary in a cell-type-dependent manner

**Fig. 4:**
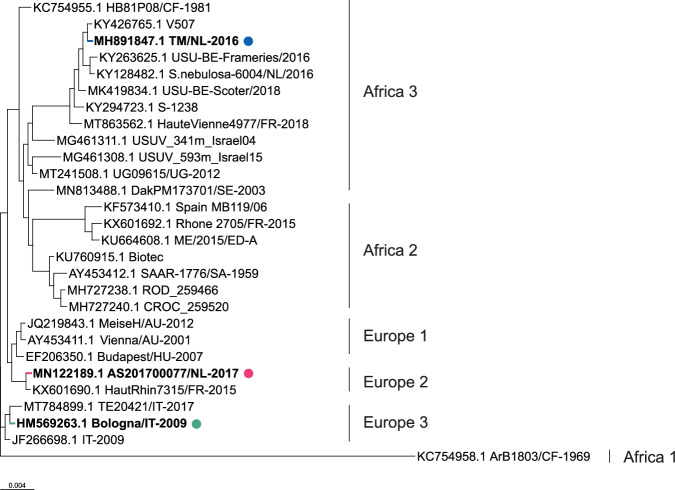
Phylogenetic tree of USUV lineages showing the positions of the strains used in this study. Phylogenetic tree of selected isolates from different USUV lineages previously used in animal studies or commonly used reference strains. Isolates used in this study are shown in bold. Polyprotein sequences of USUV viruses (3434 aa long for all presented viruses) were obtained from GenBank entries with the indicated accession numbers, and multiple amino acid sequence alignment was generated using MAFFT with default parameters^39^. The phylogenetic tree was created from this alignment using FastTreeMP with default parameters^40^ and the tree was rooted using KC754958.1 as an outgroup.

**Fig. 5:**
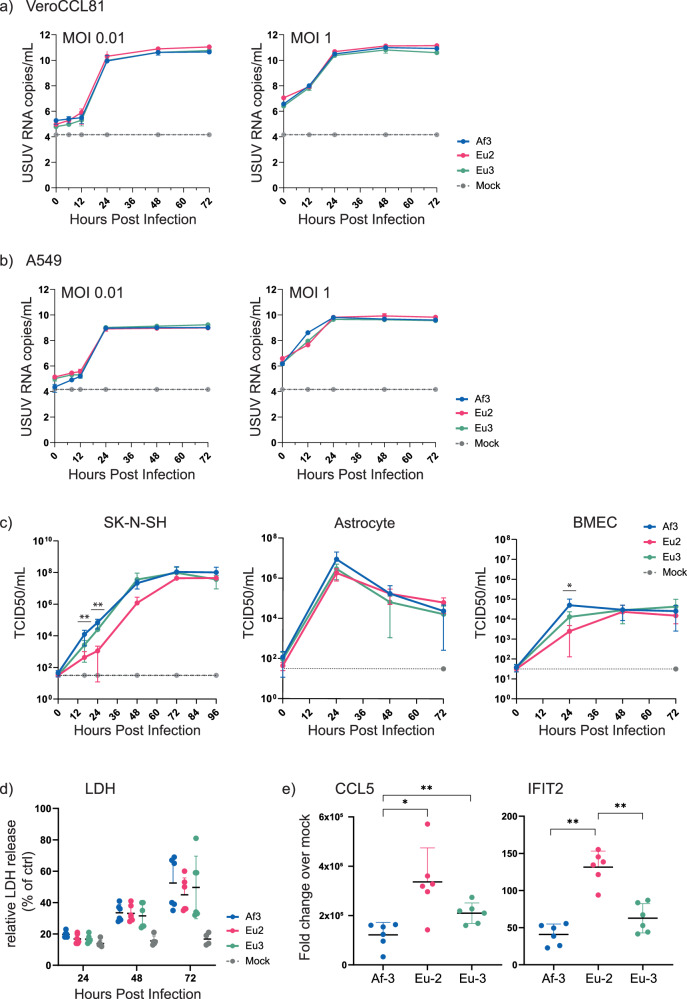
USUV strains from different lineages have similar replication kinetics in a range of different cell types. Replication kinetics of USUV strains Eu-2-IT, Eu-3-NL and Af-3-NL in **a** VeroCCL81, infected at MOI 0.01 and MOI 1, **b** A549, infected at MOI 0.01 and MOI 1, **c** SK-N-SH, MOI 0.01, Primary Astrocytes, MOI 1 and Primary BMECs, MOI 1. Viral RNA copy numbers or infectious virus titres (mean ± SD) were determined by analysis of the culture supernatant by RT-qPCR (representative result of three independent *n* = 3 experiments is shown) or TCID50 assay (results incorporate two replicate *n* = 3 experiments). Limit of detection represented as dotted grey line. **d** Virus-induced cytotoxicity as measured by the quantification of LDH release and normalised to triton-x100 lysed cells (100% LDH release); results incorporate two replicate *n* = 3 experiments. **e** Host responses to infection were assessed by quantification of the induction of CCL5 and IFIT2 expression (compared to mock-infected cells) by RT-qPCR (results incorporate two replicate *n* = 3 experiments). Statistical analysis was performed using unpaired t-test. * *P* < 0.05, ** *P* < 0.01.

To evaluate the relative virulence of different USUV strains, we compared a single isolate from three USUV lineages - Af-3, Eu-2 and Eu-3 (Fig. 4a). Af-3 and Eu-3 were selected based on their contribution to outbreaks in the Netherlands, and Eu-2 was selected for its higher pathogenicity and clinical relevance. We first compared replication kinetics in Vero CCL-81 cells, and found no differences between the three strains at either high or low MOI (Fig. 5a). We next compared growth kinetics in A549 cells, which are also susceptible to USUV infection but, in contrast to Vero cells, have an intact innate immune response^27^, and also found no differences between the strains (Fig. 5b).

Due to the neuropathogenicity of USUV we also compared replication of these strains in SK-N-SH cells, a neuroblastoma cell line with epithelial morphology. In addition, we have previously shown that USUV Af-3-NL can replicate in cells of the human blood-brain barrier—astrocytes and brain microvascular endothelial cells (BMECs)^28,29^. Here, we compared replication of two additional lineages with Af-3. In the astrocytes we observed no differences in replication kinetics between the USUV strains, however, in contrast to what we expected from the literature^17–19^, in the SK-N-SH and BMEC cells Eu-2-IT replicated slower while this strain did reach similar peak titres at a later time point (Fig. 5c). To further assess potential differences between these strains we measured lytic cell death and host responses in infected A549 cells. USUV infection caused cell death as indicated by the release of LDH from cells into the medium (Fig. 5d), but we observed no statistically significant difference between the various isolates. The host innate immune and inflammatory responses to infection were assessed by measuring changes in mRNA expression of a chemokine, CCL5, and an interferon-stimulated gene (ISG) IFIT2, using cell lysates harvested at 24 hpi (Fig. 5e). Relative expression levels of both immune genes were 2–3 fold higher in the Eu-2 infected cells than in Af-3 or Eu-3 infected cells; in the Eu-3 infected cells, expression levels were higher than in the Af-3 infected cells, although a statistical difference was observed only for the expression of CCL5. This suggested that despite the lack of difference in observed pathogenicity, there was a variable innate immune response induced by the different isolates.

### USUV Af-3-NL is less pathogenic in mice than strains from other USUV lineages

**Fig. 6:**
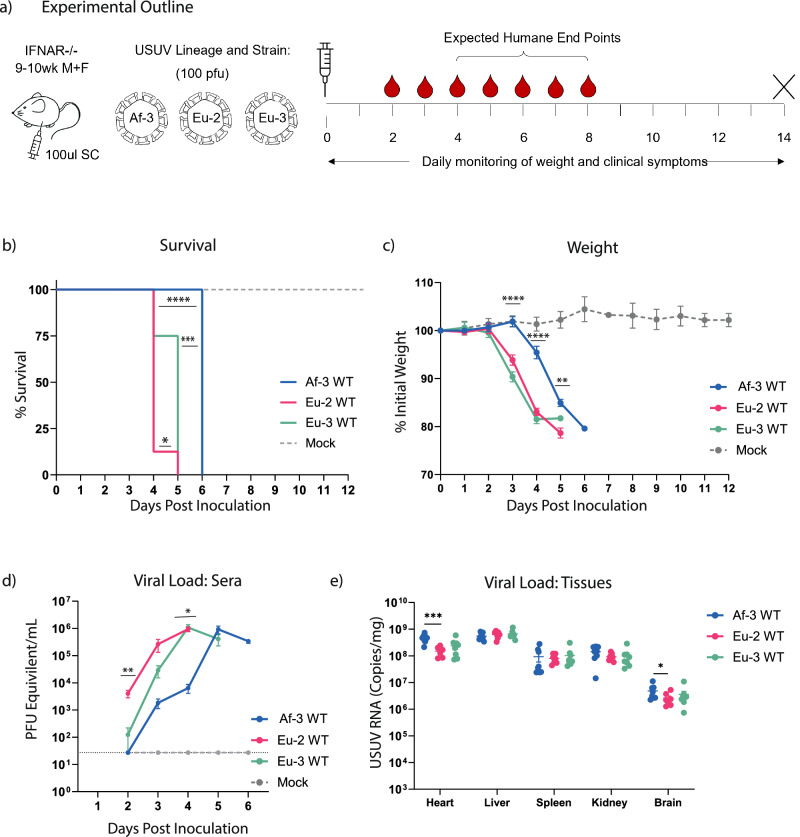
An USUV Europe-2 strain exhibits accelerated lethality compared to the Africa-3 Netherlands strain in an Ifnar^−/−^ mouse model. **a** Ifnar^−/−^ mice were inoculated subcutaneously (SC) with 1 × 10^2^ pfu/mouse of Eu-2-IT, Eu-3-NL or Af-3-NL (*n* = 8) or with DMEM (*n* = 3), weighed daily and half the mice from each group were tail bled on alternate days. Animals were euthanized when they reached humane endpoint, final bleeds were taken by heart puncture and relevant tissues were harvested. **b** Survival rates for each of the experimental groups. Statistical analysis was performed using the log-rank (Mantel-Cox) test. **c** Daily weight loss measured as a percentage of initial weight for each of the experimental groups showing mean ± SD. Statistical analysis was performed using a one-way ANOVA for each time point. **d** Mean (±SD) viral load measured by RT-qPCR of tail bleeds and final heart bleed sera, using serial dilutions of a reference standard to determine pfu equivalents. Statistical analysis was performed using a one-way ANOVA for each time point. **e** USUV RNA copies/g of homogenised heart, liver, spleen, kidney and brain tissues harvested at humane end point or end of experiment (day12) measured by RT-qPCR and absolute quantification using a reference standard. Statistical analysis was performed using unpaired t-test corrected for multiple analysis. Limit of detection represented as dotted grey line. *P < 0.05, **P < 0.01, ***P < 0.001, ****P < 0.0001.

To compare replication kinetics and pathogenesis of the USUV strains from the different lineages in vivo, we administered 100 pfu of either Eu-2-IT, Eu-3-NL, or Af-3-NL subcutaneously into the hind limb of mice as in earlier animal experiments (Fig. 6a). The results obtained with Af-3-NL-infected mice replicated our earlier observations for the 100 pfu dose, where 100% of the mice succumbed by day 6 post-inoculation (Fig. 6b). Mice infected with Eu-3-NL and Eu-2-IT, however, exhibited accelerated disease progression, including earlier onset of weight loss, leading to lethality by day 5 and day 4 respectively. Similar to our observations using different USUV doses, viremia closely mirrored weight loss, peak viral titres were similar across all strains, and onset of symptoms preceded lethality by approximately 2 days (Figs. 6c, d). We also assessed viral load in tissues harvested at time of death, and found that Af-3-NL achieved a marginally higher titre than the other strains in most tissues, with significant differences observed in the heart and forebrain (Fig. 6e), possibly due to a prolonged replication period within the animal compared to the Eu-3-NL and Eu-2-IT strains where lethality occurred more rapidly.

## Discussion

In this study we seek to better understand the lethality of USUV in a mouse model. We compared the virulence of 3 different strains isolated during European outbreaks and characterised an Ifnar^−/−^ mouse-based infection model using low doses of virus. We determined a dose that increased the average survival time to better facilitate comparative studies of USUV isolates and to enable future efficacy studies for vaccines or antivirals.

The use of doses of 10^3^ pfu/mouse or higher, which have been described in several other studies (Table 1), causes very rapid lethality. We found that an infection dose as low as 20 pfu of USUV Af-3-NL per mouse was sufficient for a lethal model, and that the time from onset of symptoms (weight loss, limp in injected limb) until HEP was longer at this low dose.

The occurrence of lethality that we observed at such a low dose, as well as the rapid lethality we observed at higher doses, is consistent with results from other studies on Ifnar^−/−^ mice infected with USUV from various lineages (Table 1). For comparison, closely related viruses WNV and JEV are lethal within 3–5 days at doses as low as 1 pfu/mouse^30^. The Af-3-NL strain, however, had previously been shown to be non-lethal in this model, which sharply contrasts our observations. In these earlier studies, adult Ifnar^−/−^ mice infected with 10^3^ pfu of the Af-3-NL strain displayed 90% survival, while mice infected with other Af-3 lineage viruses all succumbed to the infection^16^. Af-3-NL was also less virulent in two other models; weanling Ifnar^−/−^ mice, and CD-1 mice made transiently susceptible by injection of an anti-IFNAR antibody^11^.

To exclude that the discrepancy between our and the published results depended on an underestimation of the titre of our virus stock, we confirmed the titre by different techniques in two independent laboratories. By comparing the sequence of our Af-3-NL isolate with that of the published studies, we found only a single amino acid difference between them, E293K in the Envelope protein. We introduced the E293K mutation in our Af-3-NL isolate using reverse genetics and compared it in the mouse model to the wild-type isogenic control. The introduction of the E293K in our Af-3-NL isolate did not result in increased survival of the mice.

Another difference between our study and that by Kuchinsky et al. is the site of injection, since we injected in the hind limb rather than the footpad, and the exposure of various skin cell types to the virus may differ between these injection sites. However, Kuchinsky et al. also assessed other USUV strains (from both Af-2 and Af-3 lineages) alongside Af-3-NL, and these demonstrated lethality by day 6, similar to our results with other strains^16^. We therefore suspect that the difference in inoculation site is unlikely to explain our contrasting results, unless the location of injection impacts the virulence of Af-3-NL strain specifically but not that of the other Af-3 lineage strains. While we did observe that in Af-3-NL-infected mice disease progression was slower compared to mice infected with the other USUV lineages that we tested, in line with other studies, the striking difference between our observations and those of Kuchinsky et al. remains unexplained^17^. Future (collaborative) studies should provide more insight into this intriguing issue.

While studies from Kuchinsky et al. found that Af-3-NL was not lethal in Ifnar^−/−^ mice, this lack of pathogenicity was not observed in all infection models, or with any other Af-3 strains. Af-3-NL is more virulent than Af-3/UG-2012 in both mosquito and avian models^16,31,32^. In neonatal Swiss mice, an Af-3 strain (HauteVienne4977/FR-2018), though less pathogenic than the Eu-2 isolate, was more virulent than strains from several other lineages including Eu-3 (HautRhin7315/FR-2015)^17^. In 3–4 week-old Swiss mice, an Af-3 strain (CAR-1981) was similarly lethal to the Af-2 strain ROD259266, but both were less pathogenic than Af-2 SAAR-1959^33^ (Table 2).

In our in vitro lineage studies comparing Af-3, Eu-2 and Eu-3 USUV isolates, we observed no difference in the replication kinetics in Vero and A549 cells, in line with other studies^16,19^. We also observed no difference in cytopathic effect (LDH release from the host cell) of the various lineages, but did observe slight strain-specific differences in the induction of CCL5 and IFIT2, suggesting there are strain-specific differences in the innate immune and inflammatory responses. Differential expression of host genes between isolates has been observed previously in brain tissue of infected mice and in primary human astrocytes^17^. We did not see differences in replication kinetics between the three strains in primary astrocytes but observed decreased replication of Eu-2-IT in both SK-N-SH and primary BMECs. This contrasts with other studies in primary brain/neuronal cells, and in primary keratinocytes, where strains from a range of lineages had different replication kinetics and grew to different titres, with the Eu-2 isolate (TE20421/IT-2017) consistently replicating more efficiently^17,18^ (Table 2). Enhanced pathogenicity of the Eu-2 (isolate TE20421/IT-2017) relative to other USUV strains was also observed in vivo (neonatal Swiss mice) by the same group^17^. The Eu-2 isolate reduced average survival time and caused seizure symptoms which were not observed with other strains. While we did not observe the seizure symptoms with any of our tested USUV strains in Ifnar^−/−^ mice, we did find that Eu-2-IT caused more rapid mortality than the other strains assessed in our model. The Eu-2-IT strain also outcompeted the Af-3-NL strain in co-infection studies in Vero, DF-1 and C6/36 cell lines, as well as in a *Culex pipiens* co-infection model^19^. Furthermore, the Eu-2 TE201421 isolate and a second Eu-2 isolate, TE18982 (IT-2017), were described to cause an atypical CPE in primary human astrocytes that was not observed for other lineages, suggesting that the enhanced virulence is conserved within the Eu-2 lineage^14^. In contrast, in avian models, an Eu-2 strain (UR-10-Tm) was not more virulent^34^ (Table 2). Whether this is due to the specific isolate used or whether the enhanced virulence is specific to mammalian hosts requires more investigation.

The relative virulence of Eu-3 also appears to be model and/or isolate-dependent. In our Ifnar^−/−^ mice Eu-3-NL was more virulent than Af-3-NL but less than Eu-2-IT. However, others found that in neonatal Swiss mice Eu-3 (HautRhin7315/FR-2015) was less pathogenic than Af-3 (HauteVienne4977/FR-2018)^17^ and in wild-type mice no difference was seen between Eu-3 (BE-Seraing/2017) and Af-3 (BE-Grivegnee/2017) strains. In wild birds and experimentally infected chicken embryos there appeared to be no difference in pathology between animals infected with Eu-3 and Af-3 lineage viruses although in primary avian cells, the Eu-3 isolate was more virulent than Af-3^20,34^ (Table 2).

The limited literature available and the lack of studies that replicate findings make it difficult to assess whether apparent/observed differences are model or strain-specific. Taking along a reference strain, for example, Vienna 2001 or South Africa 1959, in future studies on USUV virulence, would be useful to allow comparison of results between different studies and to better understand the lineage-specificity of the observed phenotypes. In addition to this, a limitation of our study is that we have compared only a single isolate from three selected lineages, making it hard to conclude whether the observed effects are lineage- or only isolate-specific. Including additional strains from the same lineage and incorporating a broader range of the different USUV lineages would provide a more comprehensive overview of phenotypic variation between the USUV lineages.

We acknowledge that the use of Ifnar^−/−^ mice as an infection model is not ideal given its defects in innate immune response, which alters the pathology of the virus (which would normally be restricted by the host response) and impairs adaptive immunity (making these mice less ideal for antiviral or vaccine studies)^30^. However, wildtype mice are not reliably susceptible to the virus and the alternative—the use of very young animals—also might not model human infection very well^9,35^. On the other hand, severe manifestation of USUV infection is mainly observed in immune-compromised humans and therefore the use of Ifnar^−/−^ mice might offer a representative model to study the occasionally severe USUV pathogenesis in humans. Currently in vitro infection models that allow the study of pathogenesis, and that more accurately reflect strain-specific differences in virulence, are lacking. The use of mosquito and avian infection models is not an alternative for mouse studies, as strain-specific phenotypes in these models were found to differ from those in mammalian models^9,19,31,32,34^.

The inter-species transmission dynamics and the impact of environmental factors mean that integrating human, animal, and environmental health considerations in a One Health approach is essential for a comprehensive understanding and effective management of USUV.

Here we further describe a relevant and well-characterised mouse model to study USUV pathogenesis and compare three different USUV strains using this model. Understanding USUV replication and pathogenesis will help to better predict the human health risks associated with circulating and emerging strains of the virus including any novel variants.

## Supplementary information

Duyvestyn et al Lethality of Usutu isolates_Supplementary Information_Revised_3

## Data Availability

No datasets were generated or analysed during the current study.
