# Supplementary material for: Dose and strain dependent lethality of Usutu virus in an Ifnar−/− mouse model
Source: Npj Viruses. 2025 Jan 28;3:6. doi: 10.1038/s44298-025-00089-x (PMC11775335; doi:10.1038/s44298-025-00089-x)
Supplement: Supplementary file 1 — Duyvestyn et al Lethality of Usutu isolates_Supplementary Information_Revised_3 [file 44298_2025_89_MOESM1_ESM.docx]

Supplementary Figure 1.


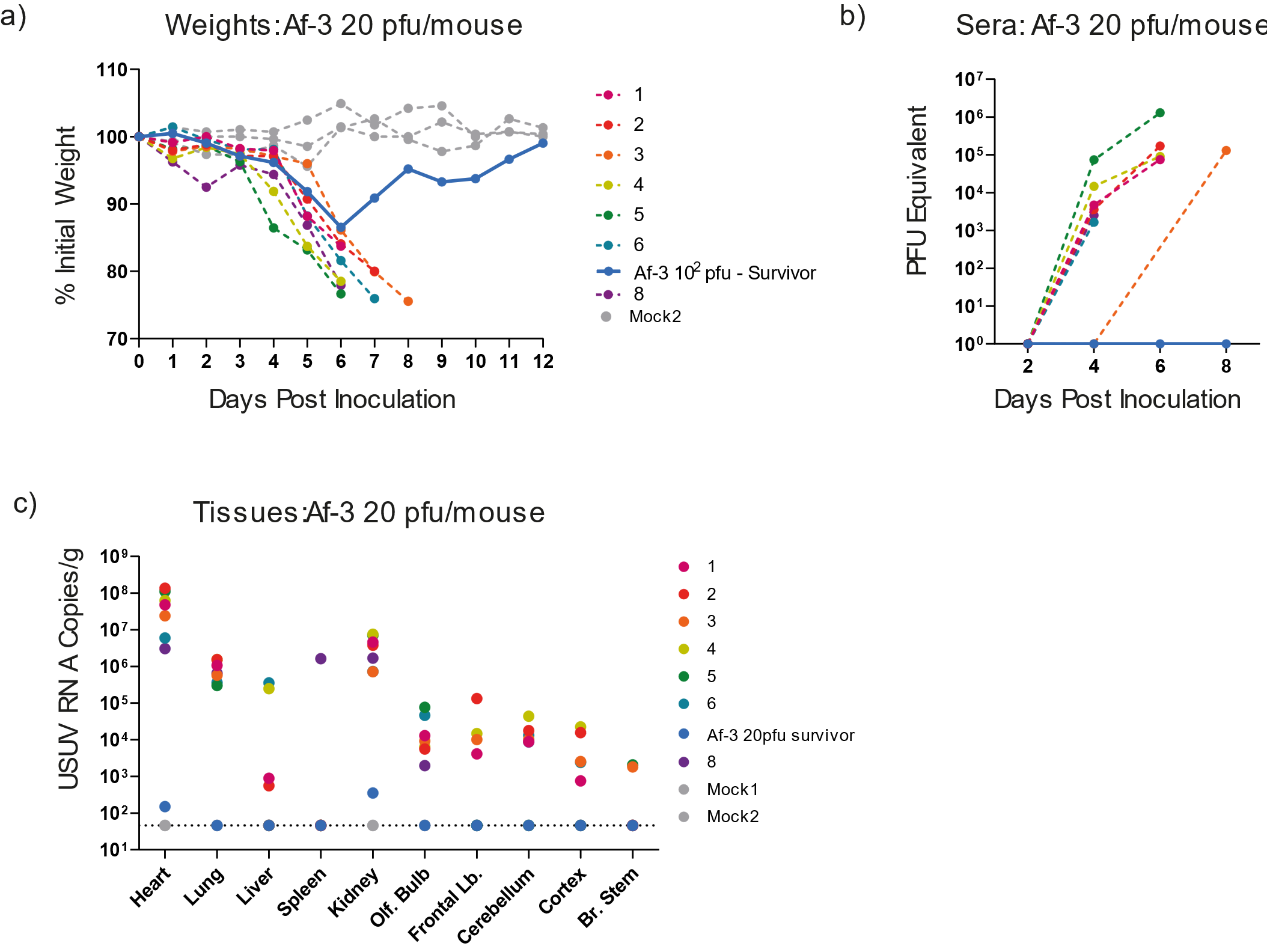


**Supplementary Figure 1. Individual Data for Af-3-NL 20 pfu animals**

a) Weight loss for each individual mouse as percentage of starting weight showing the recovery of the single surviving animal (shown in blue). Mock infected animals are all depicted in grey. b) Viral load of tail and heart sera for each individual mouse measured by RT-qPCR against a reference standard to determine pfu equivalents. c) USUV copies/g of homogenised tissues for each individual mouse harvested at humane end point or end of experiment (day12) measured by RT-qPCR using a reference standard.

Supplementary Figure 2.


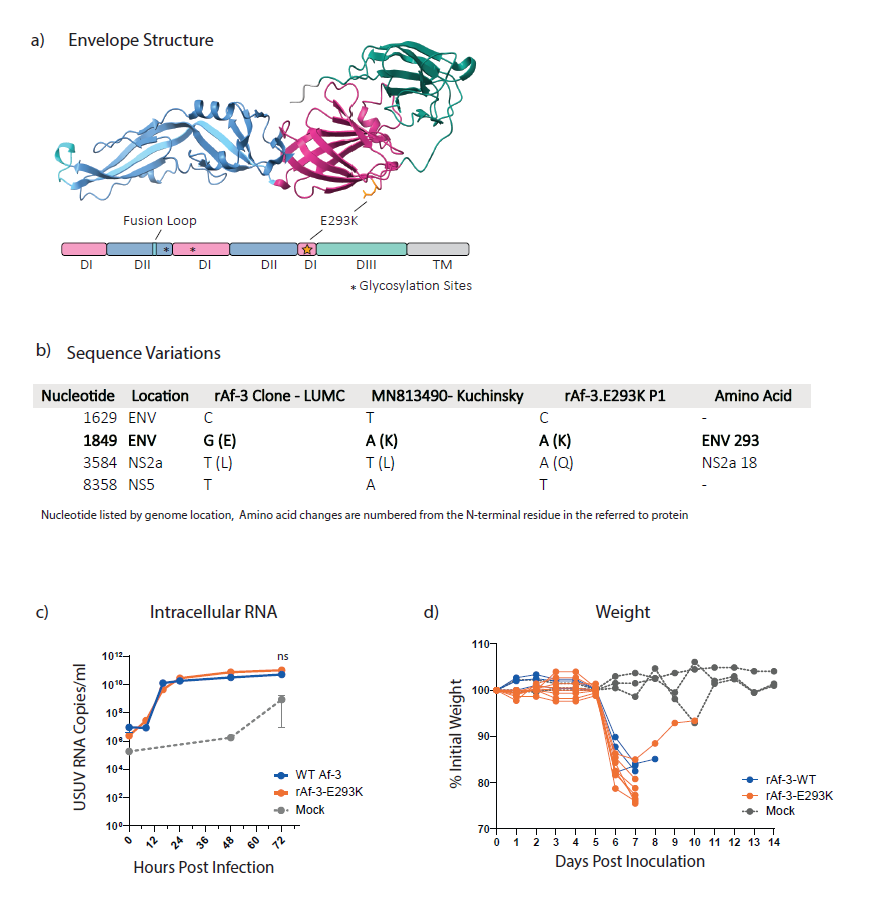


**Supplementary Figure 2. E293K mutation design and result details**

a) Cartoon protein structure of the USUV envelope (E) protein coloured by the three domains (Edited from PDB (protein data bank) structure Usutu – 6A0P (Chen, Z., Ye, F., Lin, S. et al. 2018. Crystal structure of Usutu virus envelope protein in the pre-fusion state. Virol J 15, 183.) Shown in orange is residue 293, a glutamic acid in our rAf-3-WT virus, which was mutated to a lysine to match the sequence of Netherlands 2016 (MN813490). b) Replication kinetics of rAf-3-WT and rAf-3-E293K. USUV RNA copy numbers were determined by internally controlled multiplex RT-qPCR on RNA isolated from the culture medium of VeroCCL81 cells infected with USUV at an MOI of 0.01. Statistical analysis was performed using unpaired t-test corrected for multiple analysis. c) Weight loss for each individual mouse as percentage of starting weight showing the weight gain of a single animal in the rAf-3-E293K group (orange). WT control animals shown in blue and mock animals in grey. d) Table of substitutions between USUV Africa-3 TM-Netherlands 2016 Strains and in the rAf-3-E293K recombinant virus (p1 = Passage 1).

**Supplementary Table 1: Primers used in this study**

A) Primers used in rAf-3-E293K Site Directed Mutagenesis

| **Primer** | **Sequence** |
| --- | --- |
| E293K_SDM_Fwd | GTGTCGAGTCAAGATGAAAAAGTTGACACTAAAAGGC |
| E293K_SDM_Rev | GCCTTTTAGTGTCAACTTTTTCATCTTGACTCGACAC |

B) Primers used in USUV qRT-PCR protocol

| **Primer** | **Sequence** |
| --- | --- |
| FWD_USUV_qPCR_1 | TCAGAAAAGACGTGCCAGAG |
| REV_USUV_qPCR_2 | AAAGTCCTTCCGTCCTTCATG |
| Probe_USUV_1-FAM | CCTGAAAGTGGTTTGAGCAGAAAGGC |
| FWD_417EAVs | CATCTCTTGCTTTGCTCCTTAG |
| REV_JD015 | GCTTTGCCATTGGGTTGATACC |
| Probe_615EAV-TQ-CY5 | CGCTGTCAGAACAACATTATTGCCCAC |

C) Primers used in Sybr RT-qPCR protocol

| **Primer** | **Sequence** |
| --- | --- |
| IFIT2 Forward | GGACCAAAGTCTAAATAGGG |
| IFIT2 Reverse | GGCACTTGAATTCACATTG |
| CCL5 Forward | ACAGGTACCATGAAGGTC |
| CCL5 Reverse | TGGTGTCCGAGGAATATG |
| RPL13a Forward | AAGGTGGTGGTCGTACGCTGTG |
| RPL13a Reverse | CGGGAAGGGTTGGTGTTCATCC |

**Supplementary Table 2: Titration of USUV inoculum used in animal experiments**

1. Back titrations for in vivo experiment 1 (Initial Doses)

| **Group** | **Pfu/ml: Expected** | **Pfu/ml: Measured** | **Pfu/Mouse Calculated** |
| --- | --- | --- | --- |
| 5.00E+04 | 5.00E+05 | 3.90E+05 | 3.90E+04 |
| 1.00E+04 | 1.00E+05 | 6.05E+04 | 6.05E+03 |
| 5.00E+03 | 5.00E+04 | 3.60E+04 | 3.60E+03 |
| 1.00E+03 | 1.00E+04 | 9.20E+03 | 9.20E+02 |
| 5.00E+02 | 5.00E+03 | 4.55E+03 | 4.55E+02 |

1. Back titrations for in vivo experiment 2 (Reduced Doses)

| **Group** | **Pfu/ml: Expected** | **Pfu/ml: Measured** | **Pfu/Mouse Calculated** |
| --- | --- | --- | --- |
| 5.00E+02 | 5.00E+03 | 1.70E+03 | 1.70E+02 |
| 1.00E+02 | 1.00E+03 | 3.70E+02 | 3.70E+01 |
| 2.00E+01 | 2.00E+02 | 2.10E+02 | 2.10E+01 |

1. Back Titrations for in vivo experiment 3 (E293K mutant)

| **Group** | **Pfu/ml: Expected** | **Pfu/ml: Measured** | **Pfu/Mouse Calculated** |
| --- | --- | --- | --- |
| rAf-3_WT | 1.00E+03 | 4,0E+02 | 40 |
| rAf-3_E293K | 1.00E+03 | 6,4E+02 | 63,50 |

1. Back titrations for in vivo experiment 4 (Lineage Comparisons)

| **Group** | **Pfu/ml: Expected** | **Pfu/ml: Measured** | **Pfu/Mouse Calculated** |
| --- | --- | --- | --- |
| Af3WT | 1.00E+03 | 4.80E+02 | 4.80E+01 |
| Eu2 WT | 1.00E+03 | 3.30E+02 | 3.30E+01 |
| Eu3 WT | 1.00E+03 | 2.55E+02 | 2.55E+01 |
